# Editorial: Bridging (Epi-) Genomics and Environmental Changes: The Livestock Research

**DOI:** 10.3389/fgene.2022.961232

**Published:** 2022-07-05

**Authors:** Jingyue Ellie Duan, Jicai Jiang, Yanghua He

**Affiliations:** ^1^ Department of Animal Science, Cornell University, Ithaca, NY, United States; ^2^ Department of Animal Science, North Carolina State University, Raleigh, NC, United States; ^3^ Department of Human Nutrition, Food and Animal Sciences, University of Hawai`i at Mānoa, Honolulu, HI, United States

**Keywords:** genomics, epigenomics, complex traits, environmental factors, climate changes, GWAS

Understanding the links between genetics, epigenetics, and environmental factors throughout animal development, growth, and reproduction poses new research opportunities to improve selective animal breeding, restore productivity under environmental stressors, and facilitate disease and nutrition management. Despite many emerging tools becoming available for genomic and epigenomic research, it remains challenging to connect the dots among causative genetic variables, molecular mechanisms, environmental stressors, and phenotypic variability of animal populations.

In this research topic, we covered research data that uses cutting-edge approaches to study the effects of environmental changes on animal genetics (Shen et al., meiotic recombination rates), epigenetic modification (Diaz et al., DNA methylation), and gene expression (Diaz et al., mRNA). Moreover, we covered several studies of genetic (Próchniak et al., genetic diversity) and epigenomic regulations (Shi et al., mRNA and DNA methylation; and Xu et al., microRNA) to dissect molecular mechanisms underlying complex economically important traits in livestock using next-generation sequencing approaches.

## Determining the Impact of Non-Genetic Factors on Meiotic Recombination Rate

Meiotic recombination is a fundamental biological process that facilitates meiotic division and promotes genetic diversity. Errors in meiotic recombination can result in chromosomal abnormalities that underlie complex diseases (Zheng et al., 2010). The recombination rate, estimated as the average crossover number per meiosis, has been well characterized in cattle due to the availability of large pedigrees and SNP genotype data (Ma et al., 2015; Shen et al., 2018). While most studies have been conducted in the model species, Ma et al. (2015) have first reported the genetic control of sex-specific recombination in dairy cattle. Using the large cattle genomic database maintained by the U.S. Council on Dairy Cattle Breeding (CDCB), the effects of environmental temperature and maternal age on recombination rate were investigated in North American Holstein cattle Shen et al. They used 305,545 three-generation families’ SNP genotype data and identified 6,677,618 maternal crossover events. The authors reported a U-shaped relationship between maternal age and the recombination rate, which is different from either an increasing or decreasing trend reported in humans. They also found a positive correlation between environmental temperature during fetal development of offspring and recombination rate in female parents. Based on a mixed model including maternal age and temperature, they observed the heritability of 10% for the recombination rate, demonstrating the impact of both genetic and environmental factors on meiotic recombination.

## Identifying the Effect of Environmental Heat Stress on Epigenetics

Agriculture production and livestock husbandry, particularly the dairy and beef industries, are vulnerable to climate change as higher temperatures and less rainfall will have adverse effects on cattle (Adhikari et al., 2022). Global warming has been increasing, resulting in cows exposed under heat stress. The average number of extremely hot days in the United States, already growing, is expected to more than triple from 2050 to 2,100 (Roach, 2016). Heat stress not only impacts reproductive competence and reduces pregnancy rates (Wolfenson and Roth, 2018) but also decreases immunity and increases animal infectious disease/death rates (Biffani et al., 2016), costing the US dairy industry over $1.5 billions/year (Laporta et al., 2020).

Environmental heat stress reduces the developmental competence of oocytes and embryos, which is a primary cause of sub-fertility problems in cattle (Wolfenson and Roth, 2018). The negative impact of heat stress on cow reproduction and fertility has been reported primarily at the physiological level. However, the molecular mechanisms of how heat stress affects the oocyte maturation process remained unknown. Diaz et al. investigated the effect of seasonal heat stress on bovine oocyte maturation and quality after collecting the samples in spring and summer and conducted comprehensive evaluations of gene expression profiles and global DNA methylation. In this study, a great number of differentially expressed genes in GV and MII oocytes were identified to be related to heat stress in summertime compared to springtime. Many of these genes were annotated to play vital roles of maintaining oocyte quality and maturation. The *in vitro* maturation rates of GV oocytes have no significant differences between the samples collected in spring and summer. Moreover, no significant differences in DNA methylation and hydroxymethylation were found, which might be due to the limit of the immunofluorescent staining method used in this study. Thus, methylation status of specific loci requires further evaluation with sequencing-based approaches. Overall, this work shows the effect of seasonal heat stress on both the GV and MII oocytes, which provides new insights into how environmental stressors impact aberrant transcriptomic regulation in oocytes.

## Expanding Horizons in Genomics to Dissect Complex Phenotypic Traits

Environmental factors can alter gene expression via epigenetic regulation, which ultimately affects the animal’s complex phenotypic traits. Complex traits, also known as quantitative traits, are the traits that do not behave according to simple Mendelian inheritance laws, which do not follow readily predictable patterns of inheritance. They have resulted from variation within multiple genes and their interaction with behavioral and environmental factors. And they are essential in medicine, agriculture, and evolution (Griffiths et al., 2015). Before connecting the dots between environmental factors and phenotypic traits, more research is demanding to expand the horizons in defining the causative molecular mechanisms underlying these phenotypic traits.

The genetics of quantitative traits has been studied for over 100 years, but very few of the polymorphisms that cause phenotypic variations in these traits were known until recently. Genome-wide association studies (GWAS), based on the ability to determine the genotype of an individual at thousands of single nucleotide polymorphisms (SNPs), have revolutionized our understanding of the genetics of complex traits, resulting in discoveries of thousands of associations between SNPs and complex traits (Wood et al., 2014). However, the genetic architecture of complex traits is even more complex as the spectrum of genetic mutations (e.g. the types of mutations and the number of mutations involved in a single disease) is broad and more complicated, and it is often difficult to identify a single causal genetic change since these traits tend to be polygenic. Further, although coding variants have been identified in complex phenotypic traits, most of the genetic variations mapped for complex traits tend to be non-coding and likely act by altering the control of gene expression, the stability of the RNA or protein product, or post-translational modification of the protein products, rather than altering the amino acid sequence directly (Khera et al., 2018; Polygenic Risk Score Task Force of the International Common Disease Alliance, 2021), which we call epigenetic activities including DNA methylation, histone modifications, and non-coding RNAs. Therefore, it is necessary to understand both the genomic and epigenomic contributions to complex phenotypic traits.

The dynamically changing genetic structure and environmental variance are known to contribute to the overall variability of sports performance traits in competing horses. Próchniak et al. characterized the homozygosity and genetic diversity of 1,048 warmblood horses participating in the Polish Championships for Young Horses in Show Jumping by analyzing the pedigrees of these horses. They found that the average inbreeding coefficient was at an acceptable level in 2011, but it increased in the subsequent years. They also showed that modern sport horses were derived from a small number of high-quality sires. A decreasing trend of the effective population size was also observed in the reference population, which was probably associated with the increase of inbreeding. These findings illustrate the importance of using pedigree data for monitoring inbreeding levels and mating in modern sport horses.

Crossed beak in birds is a complex trait regulated by many genes, and its heritability was estimated to be 0.1 (Bai et al., 2018a). The genetic determinants of the beak deformity have been studied at the genomic (Bai et al., 2018a, Bai et al., 2018b; Joller et al., 2018), transcriptional (Bai et al., 2014), and translational levels (Sun et al., 2019). However, little is known about the expression patterns and potential roles of DNA methylation in the complex genetic disease of crossed beaks. The genome-wide DNA methylation profiles of the normal and affected mandibular condyle of crossed beaks were explored in chicks Shi et al. More than a thousand differentially methylated genes were identified between the normal and the beak deformity chicks, which participate in bone mineralization and bone morphogenesis. Also, eleven of the differentially methylated genes were also regulated by long non-coding RNAs, in which *FIGNL1* is a vital gene in the calcification of mandibular condyle. This study is a preliminary study of epigenetics in complex chicken beak deformity traits, which provided a research basis for subsequent similar studies.

It is known that reproductive traits are complex traits with low heritabilities. The number of live births in a litter as a critical reproductive trait reflects production levels and economic benefits to a pig farm (Bakoev et al., 2020; Zheng et al., 2020). A complex transcriptional network containing coding and non-coding RNAs in the ovary closely regulates the reproductive capability of sows. The expression profiles of microRNAs (miRNAs) were explored in porcine ovaries from sows with smaller litter sizes and those with larger litter sizes Xu et al. Four hundred and eleven miRNAs were identified, and miR-183 was one of the most up-regulated miRNAs in smaller-litter-size pigs. In addition, their results demonstrated that miR-183 promoted the proliferation of granulosa cells in pig ovaries and inhibited the synthesis of estradiol while promoting the synthesis of progesterone. This study not only provides a valuable transcriptional regulatory resource for understanding the mechanisms of pig ovarian function but also reveals new clues for identifying the role of miRNAs in the determination of mammalian litter size.

## Conclusion

In brief, this Research Topic highlights the recent works in understanding how environmental factors affect genetic diversity and epigenetic modifications, and also presented various works that study molecular mechanisms underlying complex traits in livestock. We close by congratulating each of the contributing authors for their outstanding work and extend our appreciation to all of the reviewers for their time and effort to improve each submission.
